# “Right diet for the right person”: a focus group study of nutritionist-dietitians’ perspectives on nutritional genomics and gene-based nutrition advice

**DOI:** 10.1007/s12687-021-00560-1

**Published:** 2021-10-27

**Authors:** Jacus S. Nacis, Marilou R. Galang, Jason Paolo H. Labrador, Milflor S. Gonzales, Aurora Maria Francesca D. Dablo, Diana Glades A. Domalanta-Ronquillo, Victor Franco J. Alfonso, Idelia G. Glorioso, Marietta P. Rodriguez

**Affiliations:** 1 Department of Science and Technology-Food and Nutrition Research Institute (DOST-FNRI), 1631 Taguig City, Metro Manila Philippines; 2 Nutrition and Food Research and Development Division, Department of Science and Technology-Food and Nutrition Research Institute (DOST-FNRI), General Santos Avenue, Bicutan, 1631 Taguig City, Metro Manila Philippines

**Keywords:** Gene-based, Genotype, Nutritional genomics, Focus groups, Qualitative

## Abstract

Advances in nutritional genomics are intended to revolutionize nutrition practice. A basic understanding of nutritional genomics among nutritionist-dietitians is critical for such advancements to occur. As a precedent to the development and integration of gene-based nutrition advice, this study aimed to assess hospital-based nutritionist-dietitians’ perceptions of nutritional genomics. A total of ten focus group discussions (FGDs) with sixty-one registered nutritionist-dietitians (RNDs) from hospitals in the National Capital Region (NCR), Philippines, were conducted from October to November 2019. Data were collected using a pretested semistructured discussion guide, and thematic analysis was subsequently performed. Diverging perceptions about nutritional genomics were noted among the FGD participants. Five themes emerged relating to the enablers and barriers of gene-based nutrition advice: training and capacity building, the extent of information to be disclosed, cost, ethical considerations, and government support. Themes related to the desired features of the gene-based nutrition advice included being consent-driven, cost-effective, technology-oriented, and guided by standards. The results of this study suggest that training and continued learning will equip RNDs to provide nutrition advice based on genetic information. However, other factors, such as cost and ethical considerations, are critical dimensions that need to be acknowledged and addressed before integrating gene-based advice into nutrition practice.

## Introduction

The completion of the Human Genome Project was a critical step in enabling personalized medicine and personalized nutrition, the latter of which is very closely aligned with the concept of nutritional genomics. As a science that examines the effects of foods and food constituents on the expression of genes, the ultimate goal of nutritional genomics is to develop a rational means to optimize nutrition through the identification of a person’s genotype (Aruoma et al. 2019). As such, gene-based personalized nutrition recommendations combine genetic information with dietary recommendations that are tailored to the phenotype (current health status and lifestyle, among others) and genotype (genetic information) of an individual to maintain health status and counteract the risk for diseases or their comorbidities (Stewart-Knox et al. 2013; Daniel and Klein 2016).

The unprecedented progress in gene-based personalized nutrition is exemplified by attempts to provide personal health testing and monitoring technologies, such as the advent of direct-to-consumer (DTC) tests that offer dietary recommendations based on a customer’s DNA sample (Drabsch and Holzapfel 2019). With such DTC tests, also known as direct-to-consumer genetic testing (DTC-GT), an individual can obtain a set of information about his or her genetic predisposition to food-related disease or traits by collecting saliva at home without any specific medical prescription (Floris et al. 2020). Aside from saliva, sample types such as hair, buccal swabs, and blood can be collected for DTC-GT.

The translation of nutritional genomics in routine clinical practice requires that healthcare professionals understand, interpret, and communicate the complex nature of the test results in which the actual risk of developing a disease may or may not be known (Castle and Ries 2007). In addition to the regulatory issues surrounding the DTC marketing of nutritional genomics tests and the need to validate the current evidence of the interaction between genetic polymorphisms and health and nutrition, increasing the capacity of healthcare professionals to deliver gene-based nutrition services also requires careful attention. It is indeed a reality that most healthcare providers are not trained in clinical genetics and molecular testing and are not fully equipped to discuss probability and risk using genetic information (Castle and Ries 2007). Low knowledge of nutritional genomics and poor confidence in incorporating this science into practice was previously demonstrated in a series of surveys conducted in the USA, Canada, and the UK (McCarthy et al. 2008; Whelan et al. 2008; Rosen et al. 2006; Vickery and Cotugna 2005).

Nutritionists and dietitians are considered the first line of contact with the public regarding nutritional genomics, and they have been identified as prime candidates to provide advice on nutrition and genetics (Kaufman-Shriqui et al. 2020; Murgia and Adamski 2017). However, the current nutrition and dietetics curricula do not include courses related to advanced human genetics, such as discussions on omics technologies, interpretation of genetic variation information, and legal, ethical, and social aspects of genetic information (Kaufman-Shriqui et al. 2020). Education and training for nutritionists and dietitians are relevant because the basic science and evidence surrounding nutritional genomics continue to progress, because clinical practice guidelines do not yet exist for gene-based nutrition advice (Horne et al. 2021) and because the expectations for nutrition professionals are increasing as a response to the growing demand for genetic testing (Araujo Almeida et al. 2019). Such training and continuing education in nutritional genomics will provide appropriate interpretation and clinical action based on the results of genetic testing (De et al. 2019) and ensure confidence and competence among nutrition professionals (Horne et al. 2021).

In the Philippines, the system to allow clinical geneticists, fellows, and nutritionist-dietitians to counsel and manage patients using genetic information is particularly focused on nutrition therapy for inborn errors of metabolism, neurology, cancer, and other enzyme-related disorders (Laurino and Padilla 2013; Padilla and de la Paz 2013). The Commission on Higher Education (CHED) of the Philippines, a regulatory body for standards and guidelines for higher degree programs for nutrition-dietetics curricula, does not require courses on human genetics but acknowledges that graduates of nutrition-dietetics programs may venture into the field of molecular biology and genetics. Additionally, the enactment of the National System for Ensuring Newborn Screening (Republic Act 9288) facilitated the practice of genetic-metabolic dietetics in the Philippines.

With gene-based nutrition recommendations becoming an increasingly common feature in the practice of nutrition-dietetics, it is essential to obtain a glimpse of the current understanding of nutritional genomics and the possible implications of translating gene-based nutrition advice in routine clinical practice. The present study assessed hospital-based nutritionist-dietitians’ perceptions about nutritional genomics. It specifically examined their perceptions of nutritional genomics, awareness of gene-based nutrition advice, perceived enabling factors that could influence the development of gene-based nutrition advice, and desired features and impacts of such recommendations.

## Materials and methods

### Study design

This qualitative study reports results based on focus group discussions (FGDs) and is part of the Interventions using Genomics-based Strategies (INGEST) of the Enhanced Nutrition Recommendations Research Program of the Nutritional Genomics Unit of the Department of Science and Technology-Food and Nutrition Research Institute (DOST-FNRI). The program aims to develop a series of evidence-informed gene-based nutrition advice. The FGDs were conducted as part of the formative research component of the research program that aimed to explore the awareness, perceptions, knowledge, attitudes, and self-efficacy of select registered nutritionist-dietitians (RNDs) as potential implementing actors of the gene-based nutrition advice.

The study was conducted according to the guidelines provided in the Declaration of Helsinki and was approved by the FNRI Institutional Ethics Review Committee (FIERC-2019–009). Written informed consent was obtained from all the participants.

### Settings and study participants

The FGDs were conducted in Level III hospitals in the National Capital Region (NCR). In the Philippines, Level III hospitals are healthcare facilities with teaching/training facilities and accredited residency training programs in medicine, pediatrics, obstetrics and gynecology, and surgery; physical medicine and rehabilitation units; ambulatory surgical clinics; dialysis clinics; tertiary laboratories with histopathology and blood banks; and third-level X-ray facilities and equipment. The NCR refers to Metropolitan Manila and is composed of 16 cities and one municipality.

A list of public and private Level III hospitals was secured from the National Health Facility Registry and the database of the Philippine Health Insurance Corporation. Letters of invitation were distributed in selected hospitals representing the four geographical districts of the NCR. Hospital-based RNDs were purposively selected as participants because their work mostly involves providing nutrition counseling services in the clinical setting. The participating RNDs came from diverse healthcare facilities offering various services and ranging from general to specialty hospitals around the metropolitan area.

### Data collection

A pretested semistructured discussion guide developed by the Technology Diffusion and S&T Services Division (TDSTSD) of the DOST-FNRI was used to collect data from the participants. During the FGDs, the participants were asked how they perceived and understood nutritional genomics, how they foresaw the use of genetic test results for nutrition advice, and how they distinguished roles in communicating such information. The FGD participants were also asked about the perceived utility, packaging, and promotion of gene-based nutrition advice.

A total of 10 FGDs were conducted from October to November 2019. Before initiating the FGDs, all participants were informed about the purpose and procedures of the study. Each discussion lasted from 60 to 90 min, with four to ten RNDs in one session. The FGDs were facilitated by experienced and trained moderators. The moderators facilitated the discussion through a predetermined outline of questions as a guide with open-ended questions and specific probes. A documentarist was also present to record all verbal and nonverbal reactions and exchanges that transpired during the discussion. The documentation was facilitated using a structured matrix. All FGDs were conducted both in Filipino and English.

### Data management and analysis

Each of the FGDs was audiotaped and transcribed verbatim. The transcripts were reviewed for accuracy and were read and reread to initialize familiarization with the data. Member checks were performed to validate the transcripts.

Qualitative data transcribed from the discussions were manually coded, sorted, and arranged into common themes. The coding process was conducted independently by the research team members, and the results were consolidated to crosscheck codes and to set a consistent definition of codes. It was during the coding that the narratives that were delivered in the Filipino language were translated into English. The sorting of the general themes and subthemes was conducted using a collaborative approach and a recursive process. The research team conducted a series of meetings for consensus building.

Dominant themes were identified through the mapping of codes. The identification of broad themes was informed by the investigators’ a priori understanding of the subject matter, with occasional crosscheck using the discussion guide. The investigators were nutritionist-dietitians, laboratory scientists, and communication experts who intended to develop and advocate gene-based nutrition recommendations. The findings were generated from the collective analysis of all the FGDs.

## Results

The FGD participants (*n* = 61) were between 21 and 60 years old, with a mean age of 39.61 ± 11.63 years. The majority of the participants were women (87%). The participants were from twelve (12) public hospitals and two (2) private hospitals.

### Knowledge and understanding of nutritional genomics

Most of the participants had heard the term “nutritional genomics” (or “nutrigenomics”) in conferences, seminars, or online video sharing platforms, while others heard it in classroom lessons during college. Several participants were not familiar with the term, hearing about it only during the FGD.

For those who were familiar with the term, a range of concepts about nutritional genomics emerged (Table 1). The following words were used by the participants to describe their impressions about nutritional genomics: genes, DNA, genetic predisposition, cellular approach to nutrition, and “right diet for the right person.” One participant described nutritional genomics as being “related to disease prevention that could delay the emergence of inherited bad genes through food consumption.”

**Table 1:** Perceptions of nutritional genomics from the focus group discussions

Dominant themes	Illustrative quotes
Genes affecting nutrition and vice versa	• “[The] nutrition of one person will depend on his or her genes and DNA” • “[The] gene affects human nutrition. The diet prescription will depend on what will be deemed bad or different. From here, we can derive the right diet for the right person.” • “It is the alignment of the diet to one’s gene.”
Genetic predisposition to a disease	• “…will find out if a person is more predisposed to cancer.” • “It is the gene-etiology of a possible disease.” • “It is the need or status of a patient based on his or her genes.”
“Cellular approach to nutrition”	• “It is in the mitochondria. It directs disease.” • “It is the cellular approach to nutrition. A type of personalized nutrition applied through technology.”
“Ethnicity”	• “It is related to ethnicity.” • “It is related to a clan, family tree, or ethnic group.”

In general, the participants understood nutritional genomics as (1) gene-environment-food interactions, (2) strategies to address noncommunicable diseases, and (3) (something) related to metabolism, heredity, and ethnicity. The participants also linked nutritional genomics with a blood type diet and inborn errors of metabolism.

### Awareness and perceptions of gene-based nutrition advice

To prime the question about gene-based nutrition advice, the moderator asked the participants about their understanding of genetics. The participants expressed that a person’s genetic makeup is the “totality of man” as it pertains to biological information and the “blueprint of an individual.”

The responses indicated that the majority of the participants had heard about gene-based nutrition advice for the first time during the FGDs. When the word “genotype” was introduced during the sessions, the participants perceived that gene-based nutrition advice was the use of genetic information in nutrition counseling. Some participants viewed gene-based nutrition advice as a genetic test that can identify the familial history of a disease as a means or strategy to prevent lifestyle diseases or as a new specialization in the field of nutrition and dietetics. Like the previously discussed understanding about nutritional genomics, several participants viewed gene-based nutrition advice as similar to recommending the so-called blood type diet, while a few linked it with human cloning.

Although the participants believed that the delivery of gene-based nutrition advice should be done in a multidisciplinary manner where doctors, geneticists, and other allied health professionals play a role, the majority deemed it important for an RND to provide gene-based nutrition advice. One participant pointed out that “since it is still nutrition counseling, then providing gene-based nutrition advice should be done by a nutritionist-dietitian.” Another participant expressed her apprehension about the possibility that gene-based nutrition advice might replace the current standards in nutrition counseling.

### Enabling factors of and barriers to gene-based nutrition advice

In the absence of prior information about gene-based nutrition advice, the majority of the participants inferred that such targeted recommendations could help RNDs and patients to understand the “effect of food on them and why such effects are inherited.” They assumed that the advice would help RNDs recommend a better and more specific diet. In the course of the FGDs, several factors that might influence RNDs’ willingness to adopt the gene-based nutrition advice emerged (Table 2). Dominant themes included the need for comprehensive training for RNDs (who would deliver the advice) and for laboratory professionals such as medical technologists (who would perform the genetic testing), the extent of information that should be provided to patients, the importance of factoring the cost or expense a patient might incur in following gene-based nutrition advice, and the ethical implications of a genetic test.

**Table 2:** Perceived enabling factors of and barriers to gene-based nutrition advice

Dominant themes	Illustrative quotes
Training and capacity building	• “I need to study more. [I] need more knowledge.” • “RNDs can be ready if they can have the proper training.” • “Medical technologists should be well-informed.” • “Since doctors are the front liners [in giving out prescriptions], they also need training.”
The extent of information to be disclosed	• “Patients will only agree if they are provided with information.” • “They need to know if this is expensive or if this is free. [To let them know] what are the potential benefits, the advantages or disadvantages [of the advice].”
Cost	• “As long as the facility is available. However, there is an issue with the cost. The patients will prioritize their medicines.” • “Will the effectiveness and accuracy justify the cost?” • “How affordable are the [genetic] tests?”
Ethical considerations	• “The result may show that I am an adopted child.” • “Must go through ethics [approval] first before implementation.”
Government subsidy and support	• “The challenges include the acquisition of the technology [needed to test the DNA], standardization [of methods and guidelines], the patient’s financial capacity, and the need for the support of the Department of Health.” • “[I] need a directive from the Department of Health for it to be done.”

The emergence of a theme related to subsidies and government support during the FGDs was inferred to be significant given the current context of healthcare in the Philippines. One participant, for example, pointed out that patients would prioritize buying their medicines before following gene-based nutrition advice. Some participants also emphasized that an administrative order from the Department of Health (DOH) of the Philippines mandating the practice of gene-based nutrition advice could be an important recognition of this novel nutrition and health intervention.

The provision of formal training on nutritional genomics for nutritionist-dietitians is also of particular interest among the dominant themes that emerged during the FGDs. Most of the participants thought that a comprehensive understanding of the relationship between genomics and nutrition is the missing link in the effective delivery or translation of gene-based nutrition advice, as genetics is barely addressed in the undergraduate curriculum of the nutrition and dietetics degree in the Philippines. When asked about their desired topics for formal training, the participants wanted to know about a basic overview of genomics, the procedures in providing gene-based nutrition advice, the interpretation of the results of genetic tests, and the steps to generate dietary advice using genotype data. The participants perceived that incorporating genomics into the academic curriculum would be necessary to enable future-generation nutrition professionals to provide gene-based nutrition advice.

### Desired features of gene-based nutrition advice

In addition to sharing their perceptions of gene-based nutrition advice, the participants provided their recommendations regarding its important features (Table 3). A frequently mentioned suggestion was the use of mobile and electronic platforms in delivering such recommendations. The participants proposed creating mobile applications and embedding the entire recommendation system into the hospital information system: from the analysis of samples during genetic testing to the provision of nutrition advice by a healthcare professional. Several participants expressed their concern about the need for a standardized manner of providing such recommendations, and they suggested the creation of a comprehensive protocol to independently interpret the genetic test results before providing nutrition advice based on genetic information.

**Table 3:** Desired features of potential gene-based nutrition advice from focus group discussions

Dominant themes	Illustrative quotes
Consent-driven	• “There has to be consent [from the patient].” • “…will need a patient’s consent. It is important to ask for consent and [know] the willingness of the patient.” • “The consent of the patient will depend on their level of education [and understanding].”
Cost-effective	• “The issue here would be how much is the cost [of the genetic test and the advice]. The biggest factor is financial and by then, how much is the added cost?” • “The patient [will use it] if it is free.” • “If the cost will be shouldered by the patient, the usage [utilization] will be low.” • “The testing is expensive, and the processing will be lengthy.”
Technology-oriented	• “It should come as a mobile application. It is more favored by the younger generation.” • “It can be accessed online or accessible via a website.”
Guided by standards and procedures	• “There should be a guide or manual on how to interpret the [genetic] results.” • “Incorporate food models that are specific to each patient. Food restrictions should be included as well.” • “Also add interactive materials, especially for children.”

Moreover, the majority of the participants agreed that to facilitate effective gene-based nutrition counseling, the genetic test results must be compared and used along with the basic personal information of the patient, such as age, gender, religion, laboratory results, family history of disease conditions, medical history, and lifestyles, such as smoking or drinking habits.

### Perceived impacts of gene-based nutrition advice in nutrition practice

The participants gave a generally positive response about the advent of gene-based nutrition advice. Many strongly agreed that it is promising, advanced, timely, specific, and beneficial to all. According to the participants, gene-based nutrition advice would allow RNDs to provide additional information to their patients and to provide the best dietary recommendation with the addition of genetic information.

However, there were concerns about the need to establish practice guidelines. Similar to the standardized protocol that was previously mentioned, such guidelines would streamline the implementation of gene-based nutrition advice, as there would be a unified approach in delivering and providing such recommendations. In addition to the need for guidelines, the participants were also concerned about data privacy, overall acceptance, applicability, cost, and sustainability of the gene-based nutrition advice if it were to become part of routine clinical practice.

## Discussion

The goal of this study was to assess hospital-based nutritionist-dietitians’ perceptions of nutritional genomics. It is apparent that the wide range of impressions and varied understanding about nutritional genomics was dependent on prior encounters with the terms “nutritional genomics” or “nutrigenomics.” In a study among 373 registered dietitians in Canada, two possible scenarios of acquiring knowledge in nutritional genomics were proposed: exposure during academic learning and attendance of scientific conferences (Cormier et al. 2014). Similarly, our participants learned about nutritional genomics mostly in conferences, with a few of them learning about it during classroom lessons during college education. Learning is a key factor that will allow nutrition professionals to integrate nutritional genomics findings into their practice, and the best way to learn about it is through university courses, attendance of scientific conferences, and continuing professional development (McCarthy et al. 2008; Cormier et al. 2014; Abrahams et al. 2018).

The varied perceptions about nutritional genomics were expected from our participants, as it was a relatively new concept to many of them. The mixed perceptions can be attributed to their low knowledge about the discipline or perhaps to the fact that nutritional genomics is not yet within the operational realms of healthcare professionals (Weir et al. 2010). Nutrition services cover a wide range of areas—from clinical nutrition to food service management to education to health promotion and public health policy—and some of these varied fields might not require frequent encounters with newer concepts such as nutritional genomics.

Younger registered nutritionist-dietitians might also be better informed about the existence of nutritional genomics, probably because they completed or attended university courses that somehow approached this discipline. In a recent study conducted among healthcare professionals in Canada, it was evident that those who had less than or equal to 10 years in healthcare practice had greater exposure to nutrigenomics as part of their educational training (Karamanoglu and Nielsen 2020). It can be inferred from our participants that despite having a college education, their varied understanding of nutritional genomics can be attributed to the minimal basic comprehension of genetic terminologies and concepts among Filipinos previously described by Abad (2012).

Regardless of the novelty of the concept, the participants believed that the delivery of gene-based nutrition advice is within the responsibilities of a nutritionist-dietitian. From the point of view of the presumed recipient of such recommendations, the participants of a randomized controlled trial conducted among healthy Canadian adults recognized dietitians as the best providers of personalized nutrition advice (Nielsen et al. 2014). Such recognition was also demonstrated in similar studies in which the participants regarded dietitians as the most qualified health professionals to talk about nutritional genomics with their clients (Cormier et al. 2014) and as the prime professionals to provide advice on nutrition and genetics (Murgia and Adamski 2017). Dietitians are highly regarded for delivering nutritional genomics advice, as they spend considerable efforts recommending diets that can guide individuals in disease prevention (Weir et al. 2010) and possess a wide skill set to perform “one-to-one” counseling to translate information into practical solutions such as menus (Abrahams et al. 2018). Registered dietitians in effect provide similar solutions to the personalized approach of nutritional genomics and might require no new skills apart from basic comprehension of genomics (Abrahams et al. 2018).

As genetic testing is the preliminary step before the provision of gene-based nutrition advice, some argue that a genetic counselor should be the one to communicate the genetic test result to individuals who might seek such recommendations (Harris et al. 2013). The potential involvement of a genetic counselor in providing gene-based nutrition advice is due to the fact that the genetic test result is the basis for creating nutritional recommendations. In this study, however, the role of a genetic counselor was not highlighted, as it was not included in the concepts that were addressed during the FGDs.

In the FGDs, the participants discussed the potential development of gene-based nutrition advice. With the assumption that such recommendations will soon be part of routine clinical practice, the participants were asked to identify potential enabling factors of and barriers to the integration of such advice into nutrition practice. The participants gave generally positive responses about the advent of gene-based nutrition advice, which aligns with the previously reported optimism among dietitians, particularly on their perception that nutritional genomics could result in the individualization of diet prescriptions (Rosen et al. 2006).

Conversely, this study identified the need for training and capacity building initiatives as the major gaps to leverage gene-based nutrition advice. This result is not surprising, as several recent studies focusing on the competencies of dietitians have found that the lack of knowledge on genetics and genomics is a barrier to professional ownership of nutritional genomics (McCarthy et al. 2008; Whelan et al. 2008; Lapham et al. 2000; DeBusk et al. 2005). Indeed, increasing genomics education in healthcare professional training programs is an important priority, along with prelicensure training to equip future dietitians in the field of nutritional genomics (Cormier et al. 2014; Salari et al. 2013).

In the FGDs, cost and ethical aspects emerged as two important considerations. These themes became dominant when the participants were asked about the enabling factors/barriers and the desired features of gene-based nutrition advice. The cost–benefit and the ethical, legal, social, and economic implications of genetic testing have been acknowledged as important factors in low- and middle-income countries (LMICs), even in realms beyond nutritional genomics (Kingsmore et al. 2012; Thong et al. 2018). In the context of nutritional genomics, price (cost) has been negatively associated with the adoption of personalized nutrition (Rankin et al. 2018). Although the recommendation itself might be free, the issue of cost concerns the expenses related to genetic testing, which is an important prerequisite of gene-based nutrition advice. The participants of our FGDs were apprehensive that the patients might prioritize spending for their medicines and maintenance drugs rather than allotting a considerable amount of money for genetic testing. The illustrative quotes related to government subsidies and support emanated from this reality of the healthcare system in the Philippines. In addition to the actual cost for genetic testing, the participants were also cognizant about the costs needed to establish a genetic testing laboratory and the expenses that come along with the standardization and integration of genomic information in nutrition practice, such as establishing policies and legislation related to enhancing nutrition practice in the Philippines.

With the current limited legislation surrounding the provision of evidence-based nutrition information and nutritional genomics testing, a debate concerning the strength of scientific evidence supporting the marketing and use of nutritional genomics in health practice is inevitable (Kohlmeier et al. 2016; Gorman et al. 2013). These concerns are directed toward genetic testing, specifically on the collection, storage, and protection of sensitive and personal information (Burton 2015; Camp and Trujillo 2014). Our participants particularly highlighted the need for consent or agreement to help patients decide whether they agree to undergo genetic testing. Beyond willingness, our participants were specifically concerned with how the benefits, advantages, and disadvantages of genetic testing would be properly communicated to patients given the limited understanding and knowledge about genetics. Considering that nutritional genomics as a discipline is still in its infancy, the mental and behavioral aspects of providing complex information about genomics to individuals are of concern (Bates 2018).

To date, what is considered the best standard of practice with nutritional genomics is to use a team approach, where nutritionist-dietitians can collaborate with primary care physicians and/or genetic professionals to interpret the results of a genetic test and eventually develop a comprehensive, personalized care plan (Camp and Trujillo 2014). The benefits of a team-based approach were emphasized by Karamanoglu and Nielsen (2020), who underscored the role of physicians in requesting patient bloodwork following a nutrigenomics test. Ordering bloodwork is beyond the responsibility of a nutrition professional, but this important step in monitoring the progress of an individual receiving gene-based nutrition advice can be achieved through referral to other healthcare specialists, such as physicians.

### Strengths and limitations

The FGD participants represent hospital-based RNDs employed in the NCR, Philippines, but might not provide insight into the entire profession. Moreover, their views might be inclined toward the perceptions of those who work in the government sector, as the majority of the respondents were from hospitals operated by the DOH. Privately practicing RNDs may have had greater exposure and experience with nutritional genomics and/or genetic testing than their hospital-based counterparts. It is also important to note that in terms of the percentage of participants based on gender, the majority were females, and this demographic was highly dependent on the existing workforce of the participating hospitals.

As with any qualitative research, the risk of moderator bias might be inevitable. However, our facilitators attempted to minimize this risk by refraining from making any statements to the participants that might have influenced their responses. A more detailed understanding of the conflicting views was not also addressed, as there was no follow-up interview to probe the reasons for such differences in insights and opinions.

Overall, despite all the limitations, this study represents an important step toward the realization of gene-based nutrition advice in the Philippines. As an essential part of an ongoing research program that aims to create a series of gene-based nutrition and lifestyle recommendations, the insights that were gained from the FGDs will be used to package such recommendations based on the local context and understanding. Moreover, the use of open-ended questions provided participant-generated information, which is a vital knowledge base for the development of gene-based nutrition recommendations. To date, this is the first qualitative study to address gene-based nutrition advice and perceptions in nutritional genomics in the Philippines.

## Conclusion

This study aimed to assess hospital-based nutritionist-dietitians’ perceptions of nutritional genomics. Through FGDs, we examined their perceptions of nutritional genomics, awareness about gene-based nutrition advice, perceived enabling factors and barriers that could influence the development of gene-based nutrition advice, and the desired features and impacts of such recommendations. Our findings revealed that the participants have a diverse understanding of nutritional genomics. Likewise, we determined that training and pertinent genetics education will allow nutritionist-dietitians to be sufficiently qualified to integrate nutritional genomics into their practice. In light of the current healthcare context, the discussion about the integration of gene-based nutrition advice in the Philippines goes beyond the perception and understanding of those who will provide advice. Important considerations such as the cost of genetic testing, the need for policies and legislation, and the ethical and social dimensions of nutritional genomics are critical gaps that warrant further elucidation. The participants’ acknowledgement of the utility of nutritional genomics to provide an enhanced nutrition recommendation was interesting; however, the realization of such benefits requires an integrative, systems-wide approach. 
